# Coping with Secondary Traumatic Stress

**DOI:** 10.3390/ijerph191912881

**Published:** 2022-10-08

**Authors:** Maša Vukčević Marković, Marko Živanović

**Affiliations:** 1 Laboratory for Research of Individual Differences, Department of Psychology, Faculty of Philosophy, University of Belgrade, 11000 Belgrade, Serbia; 2 Psychosocial Innovation Network, 11000 Belgrade, Serbia; 3 Institute of Psychology, Department of Psychology, Faculty of Philosophy, University of Belgrade, 11000 Belgrade, Serbia

**Keywords:** coping mechanisms, secondary traumatic stress (STS), secondary trauma, secondary exposure to trauma, traumatic experience, professionals working with refugees

## Abstract

Exposure to the traumatic experiences of others can lead to *secondary traumatization (STS)*, a condition comprising trauma-related symptoms. There is a lack of evidence on efficient ways to mitigate STS among professionals working with refugees, who are secondarily exposed to traumatic content. This study examines the latent structure of coping mechanisms and explores the predictive power of coping strategies for STS in a sample of professionals working with refugees. A total of 288 participants (age: *M* = 34.01, *SD* = 10.03; 57.3% female) working with refugees completed the COPE Inventory and Secondary Traumatic Stress Scale. Factor analysis of the COPE Inventory showed that coping mechanisms are grouped around four interrelated factors—Problem-focused, Socially supported emotion-focused, Avoidant, and Passive coping—which accounted for 46.7% of the variance. The regression model showed that Avoidant coping positively predicts *negative alterations in cognition, mood, and reactivity (NACMR)* and *intrusions*, and Passive coping was positively associated with *NACMR* and *avoidance*. Problem-focused coping was related to lower *NACMR* and *avoidance*, while Socially supported emotion-focused coping was not associated with any of the STS symptoms. In total, coping factors accounted for 10.8%, 6.3%, and 4.3% of the variance of *NACMR*, *intrusions*, and *avoidance*, respectively. The study provides a foundation for programs to mitigate STS among professionals working with refugees.

## 1. Introduction

Exposure to the traumatic experiences of others can lead to *secondary traumatization* or *secondary traumatic stress* (STS), a condition that occurs as a result of helping or wanting to help traumatized individuals [1,2,3]. Secondary traumatization mimics post-traumatic stress disorder (PTSD) [4] and relies on PTSD nomenclature, referring to both exposure to traumatic content and symptomatology structure [5,6,7]. Namely, those working with people who have survived trauma can experience intrusive memories or distressing dreams related to the traumatic events that were communicated to them, negative alterations in cognition and mood, as well as alterations in arousal and reactivity associated with the client’s traumatic experiences, thus tending to avoid any stimuli associated with these events [7]. Similarly to PTSD, previous studies exploring STS’s clusters of symptoms showed evidence supporting two-factor [8], three-factor [6,9,10], four-factor [11], and seven-factor symptom-grouping [12]. Negative effects of secondary traumatization on service providers’ well-being were demonstrated in previous studies, indicating that secondary traumatization leads to more severe depression- and anxiety-related difficulties [6] and lower overall quality of life [13]. 

Following the conceptualization of secondary traumatization, previous studies were mainly focused on exploring this phenomenon among helping professions, such as health professionals and social workers, who are frequently exposed to traumatized individuals, including hospital patients, traumatized children, and victims of violence [5,13,14,15]. 

### 1.1. Coping with Secondary Trauma

Lazarus (1993) defines coping as ongoing cognitive and behavioral efforts to manage specific external and/or internal demands that are appraised as taxing or exceeding the person’s resources [16]. From this perspective, coping is defined as a process, i.e., efforts to manage stress which can change over time and can be shaped and changed by the situational context [16]. Even though all coping strategies reduce distress in the short term, some of them have been shown to be maladaptive, i.e., in the long term, neither addressing or resolving the source of distress nor reducing negative psychological outcomes, even resulting in additional difficulties [17]. There are a few conceptualizations of adaptive and maladaptive coping, depending on whether they are focused on the stressor itself or one’s reaction to it and whether they include directly addressing the problem or managing the emotional distress. Thus, some conceptualize coping as either problem-focused or emotion-focused, while others conceptualize it as approach-focused or avoidance-focused [18].

A meta-analysis of thirty-nine studies exploring the relationship between the use of different coping strategies and various psychological difficulties showed that reliance on Avoidant coping strategies was maladaptive for depression, general distress, and post-traumatic stress disorder [18]. There was no overall relationship between the use of approach coping and distress; however, there was a small but significant relationship between the use of problem/behavioral approach strategies and experiencing less distress, and a small but statistically significant association between greater reliance on approach coping and experiencing more PTSD symptoms [18].

Similarly, even though previous studies exploring strategies for reducing secondary traumatic stress did not provide unequivocal conclusions, there is a body of evidence indicating non-productive coping strategies to be related to more severe secondary traumatization-related difficulties among professionals working with traumatized individuals, such as health, mental health, and social welfare professionals [19]. Thus, it was shown that strategies including denial are associated with higher STS scores [19,20], higher emotional exhaustion [21], and burnout [22]. Furthermore, behavioral disengagement and mental disengagement were shown to be positively associated with almost all types of dysfunctional vicarious trauma beliefs [23], while self-distraction, worry, keeping to oneself, self-blame, wishful thinking, and tension reduction were associated with more severe symptoms of secondary traumatic stress and burnout [19,22]. Another non-productive coping mechanism that was shown to be positively associated with STS scores and burnout is substance abuse, including alcohol and tobacco [20,22]. Similar findings were replicated in a recent study conducted by Akinsulure-Smith and associates (2018), which showed coping strategies grouped around denial and avoidance, such as the use of alcohol or other substances, the use of humor, expressing negative feelings, the use of distraction, denial, giving up, and self-blame, to be positively and significantly associated with STS outcomes [24].

On the other hand, a body of evidence indicates the absence of a relationship between the usage of adaptive coping strategies and STS-related psychological difficulties. The exception is the use of and reliance on social support, the most frequently studied adaptive coping mechanism, which was shown to be significantly negatively correlated with STS [25,26,27]. Manning-Jones and associates (2016) found that support from friends and family is a significant negative predictor of STS [25], while there is a growing body of evidence indicating that work-related social support is negatively associated with STS, including peer support [27], supervisor support [14,20], and organizational support [28]. In addition to social support, the use of humor was shown to be associated with fewer STS symptoms [29], while positive reinterpretation was negatively related to dysfunctional vicarious trauma beliefs [23].

### 1.2. Current Study 

Bearing in mind that service providers working with refugees are faced with people who are being exposed to numerous traumatic experiences and human rights violations [30,31,32,33,34,35,36,37,38,39,40,41], as well as severe PTSD, depression, and anxiety-related difficulties among this population [42,43,44,45], the lack of studies exploring secondary traumatization among professionals working with refugees and mitigation strategies does come as a surprise. The importance of considering this group of professionals as a priority when exploring secondary traumatization has already been already recognized by some authors [6,7,24,46,47], together with the additional work-related difficulties they face, such as uncertainty, a lack of sustainable resources, and a lack of systemic support [48], which could impose additional risks for their physical and mental health [6]. Only recently a growing evidence on secondary traumatization among practitioners providing services to refugees, including resettlement, the provision of legal and psychological aid, interpretation services, etc., has emerged [4,6,13,24]; however, no definite recommendations when it comes to both risk factors and efficient interventions have yet been provided [28]. Bearing in mind the refugee crisis that has lasted almost a decade now—and the new wave of the Ukrainian refugee crisis, the duration and impact of which still cannot be predicted—the question of efficient preventive strategies, coping mechanisms, and techniques for reducing secondary traumatization among professionals working with refugees and the protection of their mental health and well-being should be recognized as a priority. 

Therefore, this study aims to examine the latent structure of coping mechanisms and explore the predictive power of coping strategies for secondary traumatization in a sample of professionals working with refugees, migrants, and asylum seekers, for whom there is a lack of much-needed evidence that could be used as a starting point for the creation of programs for the prevention and mitigation of STS.

## 2. Materials and Methods

### 2.1. Participants 

The sample consisted of 288 professionals working directly with refugees, migrants, and asylum seekers (age range: 18–69, *M* = 34.01, *SD* = 10.03; 57.3% female) employed in more than 30 non-governmental and governmental organizations and agencies providing diverse types of assistance and services to beneficiaries (legal aid, medical aid, psychosocial support, cultural mediation, etc.). Before taking part in the study, participants had been engaged in the provision of services for at least one month, and they all were employed full-time in the care and assistance of refugees. At the time of the assessment, on average, participants had worked in the refugee protection field for 36.19 months (*SD* = 70.95).

### 2.2. Measures

Coping Mechanisms [49]. The COPE Inventory assesses the frequency of use of 15 coping mechanisms, namely: *humor*—coping with stress using humor; *acceptance*—accepting the reality of a stressful situation; *planning*—thinking about how to cope with a stressor, i.e., coming up with action strategies, thinking about what steps to take and how to best handle the problem; seeking *instrumental social support*—seeking advice, assistance, or information; seeking *emotional social support*—obtaining moral support, sympathy, or understanding; *active coping*—taking active steps, in order to try to remove the stressor or to reduce its effects, which includes initiating direct action, increasing one’s own efforts, and trying to execute a coping strategy in a stepwise fashion; *positive reinterpretation and growth*—reframing the stressor in positive terms; *religious coping*—the tendency to turn to religion in times of stress; *restraint*—waiting until an appropriate opportunity to act, holding oneself back, and not acting prematurely (active and passive strategies); *substance use*—using psychoactive substances in order to cope with the stress; *denial*—refusal to believe that the stressor exists or trying to act as though the stressor is not real; *behavioral disengagement*—reducing one’s effort to deal with the stressor, even giving up on attempts to attain goals with which the stressor is interfering (helplessness); *venting of emotions*—the tendency to focus on whatever distress or upset one is experiencing and to ventilate those feelings; *mental disengagement*—using alternative activities to take one’s mind off a problem, such as daydreaming, escaping through sleep, or escaping through immersion in TV; *suppression of competing activities*—putting other projects aside and trying to avoid becoming distracted by other events in order to deal with the stressor.

COPE consists of 60 4-point scale items, i.e., 4 per coping mechanism (1—I usually don’t do this at all, 4—I usually do this a lot). The majority of subscales demonstrated sufficient reliability (α > 0.65) with a median alpha value of 0.68 (see Table 1 for details).

Secondary Traumatic Stress (STS). The Secondary Traumatic Stress Scale (STSS) [9] is the most widely used instrument for assessing the effects of secondary exposure to trauma. The STSS is a 17-item tool initially developed to measure the negative effects of social work practice in traumatized populations. It was initially developed relying on the DSM-4 conceptualization of PTSD symptoms, measuring three symptom clusters: intrusion, avoidance, and hyperarousal. However, it has been shown that the composition of items forming the subscales of negative alterations in cognition, mood, and reactivity (NACMR); intrusions; and avoidance associated with indirect exposure to traumatic experiences has the best fit [6]. Participants are instructed to indicate how often they have experienced secondary trauma symptoms (e.g., *I thought about my work with clients when I didn’t intend to*; *My heart started pounding when I thought about my work with clients*) over the past seven days and provide their responses on a 5-point scale (1—never, 5—very frequently). The instrument demonstrated good internal consistency for subscales (α = 0.70–0.88) as well as total score (α = 0.91).

### 2.3. Procedure 

All procedures adhered to the Declaration of Helsinki, and the study was approved by the Institutional Review Board of the Department of Psychology, Faculty of Philosophy, University of Belgrade. Questionnaires were administrated online. Before taking part in the study, participants signed an informed consent form, and after completing the questionaries, they were offered a detailed debrief on the purpose of the study.

### 2.4. Data Analysis

The data were analyzed using IBM SPSS Statistics for Windows, version 21 (IBM Corp., Armonk, NY, USA) and IBM SPSS AMOS, version 21 (IBM Corp., Armonk, NY, USA). First, the descriptive measures for all variables included in the study were calculated (means, standard deviations, standardized skewness and kurtosis, and alpha coefficients). To investigate the zero-order correlations between the coping mechanisms and STS, Pearson correlation coefficients were calculated. The latent composition of coping mechanisms was examined using the Maximum Likelihood method of extraction, and the obtained factors were rotated using oblique rotation (Promax). Both Guttman–Kaiser and scree criteria were consulted to decide on the optimal number of latent dimensions of coping mechanisms to be retained. Finally, to examine the unique contribution of the dimensions of coping mechanisms to the prediction of the three aspects of STS, a regression model was tested using IBM SPSS AMOS 21 software (IBM Corp., Armonk, NY, USA).

## 3. Results

The descriptive indices (Table 1) showed positive asymmetry in the distributions of scores for the three secondary trauma subscales as well as the total score, indicating a higher grouping of scores in the lower range. The same asymmetry was obtained for the coping mechanisms of *denial*, *religious coping*, *behavioral disengagement*, and *substance use*, while *restraint* and the *suppression of competing activities* showed negative asymmetry, i.e., the were skewed towards higher scores. All three subscales of STSS, as well as the majority of the coping mechanism measures, showed good internal consistency, while some of the COPE subscales demonstrated very low reliability.

Table 2 presents the relationships between each coping mechanism and the secondary traumatic stress subscales and total score. In general, less useful coping mechanisms proved to be more predictive of secondary trauma symptoms than more useful ones. Namely, only *planning, instrumental social support*, and *positive reinterpretation* showed negative correlations with any aspect of secondary traumatic stress, while the *suppression of competing activities* was positively correlated only with intrusions. On the other hand, *substance use*, *behavioral disengagement*, and *mental disengagement* were positively associated with each of the secondary trauma symptoms. *Denial* and the *venting of emotions* were positively associated with NACMR and intrusions but not with avoidance symptoms. *Religious coping* was positively correlated with intrusions, while *humor* was positively related to NACMR (marginally *p* = 0.051). Finally, the *use of emotional social support*, *restraint*, *acceptance*, and *active coping* were not predictive of any aspect of secondary trauma.

To examine the latent structure of coping mechanisms, 15 subscales of the COPE Inventory were factorized using exploratory factor analysis (EFA). Bartlett’s test of sphericity [χ^2^_(105)_ = 1312.40, *p* < 0.001], as well as the Kaiser–Meyer–Olkin measure of item sampling adequacy (*KMO* = 0.76) showed the suitability of the correlation matrix for factorization. The Guttman–Kaiser and scree criteria suggested the retention of four latent dimensions accounting for 46.7% of the variance in coping mechanisms. The results of the *EFA* are presented in Table 3.

Four coping mechanisms were primarily loaded on the first factor—*planning*, *active coping*, the *suppression of competing activities*, and *positive reinterpretation and growth*. None of these coping strategies demonstrated substantial secondary loadings. Since this factor effectively summarized problem-oriented dealing with stressors, it was labeled Problem-focused coping. *Instrumental* and *emotional social support* both loaded highly on the second factor alongside the *venting of emotions*. Apart from dominant loading on the second factor, *instrumental social support* demonstrated secondary loading on the first factor, as well. In line with its composition, the second factor was named Socially supported emotion-focused coping. The third factor incorporated less useful coping mechanisms, summarizing typical avoidance coping mechanisms—*denial*, and *mental* and *behavioral disengagement*. In addition, *religious coping* showed relatively high and exclusive loading on this factor, as well. In line with the predominant content, this factor was interpreted as Avoidant coping. Finally, the fourth factor was composed of both useful and less useful coping mechanisms of *acceptance*, *humor*, *substance use*, and *restraint*, which all had primary loadings on this factor. In addition, *mental disengagement* showed secondary loading of nearly equal magnitude on this factor, while *restraint* demonstrated secondary loading on the factor of Problem-focused coping, as well. Since all coping mechanisms exhibiting primary loadings on this factor were neither problem- nor emotion-focused and were not characterized by avoidance in the same sense as the mechanism grouping on the third factor—but rather, were defined by dealing with the problem in a more indirect and/or passive way—this dimension was labeled Passive or Indirect coping.

Table 4 presents relationships between the extracted factors. Problem-focused coping was positively related to Socially supported emotion-focused coping, and both were positively associated with Passive coping. On the other hand, Avoidant coping was significantly and positively correlated with Passive coping only.

**Figure 1:**
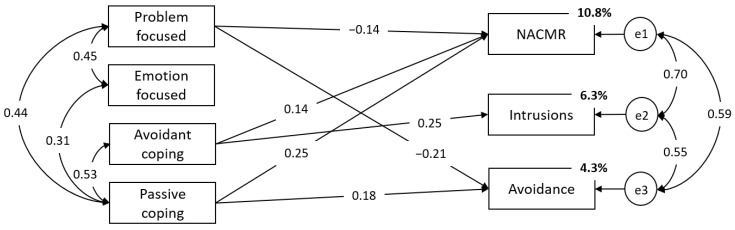
Relations between Problem-focused, Socially supported emotion-focused, Avoidant, and Passive coping with STS.

To explore the predictive power of the dimensions of coping and their exclusive relations with secondary traumatic stress symptoms, a regression model was tested. Within both sets of variables, covariations between subscales were specified based on their zero-order correlations, while regression paths were freely estimated. The results showed that Avoidant coping positively predicted NACMR and intrusion symptoms, while Passive coping was positively associated with NACMR and avoidance. Problem-focused coping was related to lower avoidance and NACMR, while Socially supported emotion-focused coping was unrelated to any of the secondary trauma symptoms. In total, coping factors accounted for 10.8%, 6.3%, and 4.3% of the variance in NACMR, intrusions, and avoidance, respectively. The prediction model with nonsignificant regression paths set to zero is presented in Figure 1 [χ^2^(8) = 8.41, *p* = 0.395, *CFI* = 0.999, *TLI* = 0.998, *RMSEA* = 0.013 (90%CI: 0.000–0.071)].

## 4. Discussion

This study aimed to examine the latent composition of coping mechanisms measured using the COPE Inventory and to determine their overall and specific contributions in predicting symptoms of secondary traumatization in individuals working with refugees, migrants, and asylum seekers. 

Regarding the latent composition of coping mechanisms, the results revealed four latent dimensions of coping, partially corresponding to those found in previous studies [49,50,51,52]. The factor of Problem-focused coping predominantly summarized approach-oriented mechanisms, characterized by cognitive engagement in dealing with the source of stress, more specifically, analyzing ways and means of managing the problem at hand. This factor has been shown to underlie the coping mechanisms of *planning*, *active coping*, the *suppression of competing activities*, and *positive reinterpretation and growth*. Although *positive reinterpretation* is occasionally considered an emotion- and not a problem-focused strategy [50], our results showed that its cognitive aspect is perhaps more salient than its emotional aspect.

The composition of the second factor, labeled Socially supported emotion-focused coping, proved to be very much in line with previous studies, as well [49,50]. Coping mechanisms that defined the second factor, namely *instrumental social support*, *emotional social support*, and *venting*, pointed to a predominant focus on distressing emotions and relying on available social support. So, this dimension of coping proved to be characterized by expressing and sharing negative emotions and experiences and relying on social support as means to manage them.

*Denial*, *behavioral*, and *mental disengagement*, as examples of less useful coping, were shown to be accompanied by *religious coping*, forming the dimension of Avoidant coping. The finding that religious coping goes hand-in-hand with less useful coping mechanisms indicates that turning to religion in times of stress potentially derives from withdrawing from the stressor and/or associated emotions, or a lack of capacity to tackle the stressor instantly, and just relying on help beyond one’s control, perhaps even reflecting one’s helplessness in managing the stressor. Bearing in mind that much of the previous work defined *religious coping* as an emotion-focused coping style [50], these findings seem to shed new light on the properties of turning to religion as a coping mechanism.

The coping dimension labeled Passive or Indirect coping, although moderately related to Avoidant coping, stood out as a relatively distinct dimension, including *acceptance*, *humor*, *restraint*, and *substance use*. Although the first three can be considered as emotion-focused coping mechanisms [50], the feature that differentiates them from both Socially supported emotion-focused and Problem-focused coping is the lack of directed action towards dealing with either the problem or the emotions induced by it. Rather, it seems that these coping mechanisms address the problem or associated emotions more indirectly and passively. Namely, mere *acceptance* of a stressor does not *per se* imply engagement in attempts to deal with it. Similarly, making light of the problem could serve as an indirect and relatively passive way of coping with the problem since it calls into use only occasional efforts to view or even just present the problem to others from a more positive perspective. Additionally, *using substances* to reduce distress operates similarly—by putting minimal effort into dealing with stress, i.e., it serves as an “easy way out” in managing the stress. Finally, although *restraint* is usually considered an active coping strategy in the sense that the behavior is oriented towards effectively dealing with the stressor, it can be a passive strategy, as well, since employing it can mean not acting [49] or even indefinitely postponing directed action. The splitting of this particular coping strategy between the first and the last factor seems to highlight its duality very well.

Regarding the relationship between coping mechanisms and secondary traumatization, the results showed that the extracted dimensions of coping differentially predicted the severity of STS symptoms. Namely, Problem-focused coping proved to be primarily related to less severe symptoms of avoidance but less pronounced NACMR, as well. These effects are primarily derived from mechanisms of *positive reinterpretation and growth* and *planning*, as only Problem-focused coping mechanisms that were predictive of any of the STS symptoms. Thus, these two coping mechanisms seem to be the main protective agencies for STS. Similarly, previous studies have shown that *positive reinterpretation* is negatively associated with dysfunctional vicarious trauma beliefs [23]. Since this coping is defined as “positively reframing the stressor”, it is the only strategy that undoubtedly includes facing the stressor itself and trying to reduce its effects in a way that is entirely independent of external factors. In the circumstances in which service providers working with refugees perform their job—which often include a lack of possibilities to influence the absence of systemic protection for their clients, traumatic experiences that have already happened and will potentially happen in the future, and the outcomes of the asylum decisions that are followed by a severe experience of helplessness [7,13,47]—it could be expected that in the majority of situations, the use of a coping strategy that is independent of external factors is the most adaptive response. Additionally, our results showed that this strategy is the only adaptive coping mechanism that is negatively related to symptoms of intrusions.

Contrary to previous findings showing that social support can act as a protective factor for STS-related difficulties [25,53], the present study found no effect of Socially supported emotion-focused coping on any STS domains. Namely, despite the *venting of emotions* being positively associated with higher NACMR and intrusions, and seeking *instrumental social support* being inversely related to avoidance symptoms, on a factor level, this dimension of coping demonstrated zero predictive value for any of the STS domains. Similarly, the only previous study exploring the relationship between coping and STS among professionals working with refugees also found social support not to be predictive of STS [24]. It could be argued that these findings are specific to secondary traumatization in the context of refugee protection. Namely, it could be assumed that multiple traumatic experiences—often including torture, the death of the loved ones, and a lack of systemic solutions and perspectives, which are a regular part of working with refugee populations—are so beyond everyday life experiences that professionals feel as if no one outside of the context could understand them, and that these experiences, if shared, could be a burden for their loved ones. Therefore, these work-related experiences might be left out of regular social interaction and not “worked through” via this means of support. This could also be a reason for the deeply rooted experience of isolation often recognized among professionals working with refugees [7]. However, it should be noted that a tendency to seek advice or assistance seems to have the potential to serve as a protective factor, at least in alleviating avoidance-related symptoms.

Avoidant coping stood out as the only coping dimension showing any association with intrusions. This finding is especially illustrative of the maladaptive properties of this type of coping since it shows that traumatic content that is put aside, out of one’s mind, seems to find a way into the consciousness; it might emerge in the form of recurrent disturbing dreams, memories of the clients’ traumatic experiences, or pronounced physical reactions in the presence of reminders of those experiences. Additionally, Avoidant coping mechanisms proved to be associated with increased NACMR, showing that more frequent employment of *denial*, *mental* and *behavioral disengagement*—i.e., withdrawing from stress or associated feelings, leaving them unaddressed and unprocessed—can be predictive of negative cognitive alterations, dysphoric affect, hypervigilance, and overall distorted reactivity.

Finally, Passive coping mechanisms proved to be the most prominent predictors of NACMR, but also of STS-related avoidance symptoms. It should be noted that much of the predictive power of Passive coping is drawn from *substance use*, as it proved to be the most prominent risk factor associated with all three symptom clusters of STS. The maladaptive nature of Passive coping, at least in the context of STS, is best seen in the prediction of NACMR and STS-related avoidance symptomatology, where in both cases, it “outperformed” Avoidant coping in the prediction. The finding that Passive but not Avoidant coping is uniquely related to the increase in STS-related avoidance symptomatology seems to validate the interpretation of this factor and its distinctiveness from Avoidant coping. 

In general, the obtained results add to the body of evidence showing that avoidant and Passive coping strategies, besides being predictive of various psychological difficulties including higher emotional exhaustion [21], burnout [22], PTSD, depression, and psychological distress [18], are predictive of symptoms of STS as well [19,20,21,24]. The results showed that avoidance-orientated and passive dealing with problems and their associated emotions potentially leaves professionals who are secondarily exposed to trauma in a vicious cycle of distress; this results in increased secondary traumatization-related difficulties that they are unable to cope with. Furthermore, latent dimensions of coping showed a certain degree of discriminant validity in predicting various STS symptoms, offering potential guidelines for designing programs aimed at preventing symptoms of STS, especially in populations working with highly traumatized individuals. However, it should be noted that the overall predictive power of coping mechanisms proved to be rather modest, accounting for between 4% and 11% of the variance in STS. This is certainly not surprising, bearing in mind that the severity of STS depends on multiple situational as well as dispositional factors that can trigger and exacerbate STS-related symptomatology [51,52,53,54,55] or, conversely, serve as protective factors.

When discussing the findings of relationships between coping strategies and STS, and the unbalanced predictive power of adaptive vs. maladaptive methods of coping with symptoms of STS, several methodological and conceptual issues should be considered. Namely, when trying to understand inconclusive findings of the relationship between adaptive coping and STS, it should be noted that previous studies often had too-broad and imprecise conceptualization of adaptive coping strategies. For instance, Manning-Jones and colleagues defined self-care as physical, mental, emotional, and work–life balance. Therefore, even though they found self-care to predict 11% of the variance in STSS scores [25], whether this broad conceptualization of self-care enables an understanding of which of their aspects contributed to the severity of symptoms or are of particular importance for STS can be discussed [25,56]. Similarly, one of the reasons for the lack of association between time devoted to leisure, self-care, research, and development, or between the supervision and traumatic stress scores found in other studies [57], could be found in the imprecise definition of these coping strategies, which leaves an open question about their construct validity. It could be assumed that both the type and quality of specific activities which could be part of these coping strategies can have a stronger impact on psychological outcomes than the mere frequency of usage of each mentioned coping strategy. Another reason for the inconsistencies in findings on the relationships between adaptive coping strategies and STS across studies could stem from the poor reliability of some of the adaptive coping subscales, particularly *active coping* and *restraint*. Finally, when trying to interpret the lack of relationship between adaptive coping and STS-related psychological difficulties, some authors suggested that, unlike maladaptive coping, adaptive coping might not be affecting negative psychological outcomes, but rather, boosting protective factors, including compassion satisfaction, and resilience [19]. However, more evidence is needed to support this perspective. 

The main limitation of our study, as well as of previous studies in this field, is the cross-sectional correlational design, which cannot show if the specific coping mechanism was used before or after the onset of STS symptoms—i.e., if the STS led to an increase in the use of a particular coping strategy, or if it was the other way around [20,57,58]. In addition, some of the previous studies showed trauma duration to be a significant moderator of the relationship between coping and STS, and that as the duration of the trauma increased, so did the association between the reliance on approach coping and experiencing less distress [17]. Future longitudinal studies are needed to explore if a person experiencing STS will cycle through different strategies multiple times, depending on the duration and the severity of distress, before some of these strategies result in a positive outcome [17,18]. 

Our study offers evidence on the latent structure of coping, as well as on maladaptive coping strategies that could lead to an increase in STS symptomatology among professionals working with refugees. These results can be used as a basis for the creation of preventive programs and interventions, whose effectiveness in the mitigation of STS among professionals working with refugees and other traumatized groups could be tested in future studies. As evidenced by our study, these programs should focus mainly on the reduction of maladaptive coping among professionals, grouped around avoidant and passive strategies, while future studies are needed to identify adaptive coping strategies and effective mechanisms of prevention and intervention; these could be used to mitigate STS and support the mental health and well-being of professionals caring for those in need. 

## 5. Conclusions

This study examined the latent structure of coping mechanisms and explored the predictive power of coping strategies for secondary traumatization in a sample of professionals working with refugees, migrants, and asylum seekers. The results provided both theoretical and much-needed practical implications that can be used as a starting point for the creation of programs for the prevention and mitigation of STS among service providers whose physical and mental health need to be protected, and whose work and efforts should be appreciated—especially bearing in mind the long-lasting refugee crisis worldwide, as well as the new wave of the Ukrainian refugee crisis, the duration and impact of which still cannot be predicted. 

## Figures and Tables

**Table 1:** Descriptive statistics for coping mechanisms and secondary traumatic stress.

	*M*	*SD*	*Min*	*Max*	*St. Sk*	*St. Ku*	α
**Secondary traumatic stress**							
NACMR	2.02	0.74	1.00	4.60	5.33 **	1.08	0.88
Intrusions	1.89	0.71	1.00	4.80	7.03 **	3.71 **	0.77
Avoidance	1.63	0.83	1.00	4.50	9.84 **	4.80 **	0.70
STSS	1.94	0.67	1.00	4.24	5.79 **	1.50	0.91
**Coping mechanisms**							
Acceptance	2.75	0.56	1.25	4.00	−1.16	−0.59	0.68
Active coping	2.88	0.41	1.75	4.00	0.88	0.83	0.32
Behavioral disengagement	1.64	0.48	1.00	3.50	2.97 **	−0.05	0.68
Denial	1.45	0.51	1.00	3.50	8.69 **	5.55 **	0.78
Emotional social support	2.84	0.67	1.00	4.00	−1.74	−1.10	0.81
Humor	2.31	0.80	1.00	4.00	1.51	−2.44 *	0.89
Instrumental social support	2.91	0.54	1.25	4.00	−1.40	−0.79	0.68
Mental disengagement	2.34	0.55	1.00	3.75	−0.93	−0.81	0.49
Planning	3.15	0.49	1.50	4.00	−0.88	1.09	0.77
Positive reinterpretation and growth	3.13	0.45	2.00	4.00	0.06	−0.81	0.64
Religious coping	1.68	0.87	1.00	4.00	8.08 **	0.94	0.94
Restraint	2.67	0.53	1.00	4.00	−2.19 *	1.32	0.51
Substance use	1.39	0.66	1.00	4.00	13.03 **	11.64 **	0.96
Suppression of competing activities	2.61	0.51	1.00	4.00	−2.74 **	1.63	0.62
Venting of emotions	2.30	0.64	1.00	3.75	1.15	−0.93	0.78

*Note. M*—mean; *SD*—standard deviation; *Min*—minimum; *Max*—maximum; *St. Sk*—standardized skewness; *St. Ku*—standardized kurtosis; α—internal consistency (Cronbach alpha); * *p* < 0.05; ** *p* < 0.01.

**Table 2:** Correlations between coping mechanisms and secondary traumatic stress.

	STSS	NACMR	In	Av
Acceptance	−0.02	0.02	−0.06	−0.10
Active coping	−0.02	−0.01	−0.01	−0.07
Behavioral disengagement	0.25 **	0.28 **	0.15 *	0.016 **
Denial	0.21 **	0.20 **	0.21 **	0.10
Emotional social support	−0.03	0.00	−0.05	−0.10
Humor	0.08	0.12	0.00	0.03
Instrumental social support	−0.04	−0.02	−0.01	−0.17 **
Mental disengagement	0.30 **	0.30 **	0.25 **	0.17 **
Planning	−0.12 *	−0.11	−0.08	−0.16 **
Positive reinterpretation and growth	−0.14 *	−0.11	−0.15 *	−0.17 **
Religious coping	0.08	0.05	0.15 *	0.04
Restraint	0.06	0.06	0.08	0.02
Substance use	0.29 **	0.29 **	0.19 **	0.27 **
Suppression of competing activities	0.10	0.09	0.13 *	−0.00
Venting of emotions	0.27 **	0.30 **	0.24 **	0.02

*Note.* NACMR—negative alterations in cognition, mood, and reactivity; In—intrusions; Av—avoidance; STSS—Secondary Traumatic Stress Scale total score; * *p* < 0.05; ** *p* < 0.01.

**Table 3:** Pattern matrix for coping mechanisms.

	1	2	3	4	*h^2^*
Planning	**0.94**	−0.02	−0.08	−0.16	0.79
Active coping	**0.68**	0.04	0.08	−0.05	0.46
Suppression of competing activities	**0.63**	0.01	0.19	0.11	0.52
Positive reinterpretation and growth	**0.58**	0.08	−0.14	0.10	0.45
Emotional social support	−0.07	**0.98**	−0.07	0.03	0.92
Instrumental social support	0.23	**0.66**	0.01	−0.11	0.57
Venting of emotions	−0.03	**0.42**	0.21	0.17	0.33
Denial	0.02	−0.02	**0.83**	−0.15	0.59
Religious coping	0.10	−0.01	**0.59**	−0.18	0.28
Behavioral disengagement	−0.22	0.01	**0.55**	0.22	0.48
Mental disengagement	0.13	0.05	**0.31**	0.29	0.32
Acceptance	0.18	0.00	−0.21	**0.61**	0.41
Humor	0.08	−0.04	0.02	**0.58**	0.38
Substance use	−0.20	0.05	−0.12	**0.47**	0.16
Restraint	0.35	−0.11	0.07	**0.39**	0.36
λ	3.00	2.24	1.81	2.18	

*Note.* λ —rotation sums of squared loadings; *h^2^*—communalities. The highest factor loadings for each coping mechanism are printed in bold.

**Table 4:** Factor correlation matrix.

Factor	1	2	3	4
1. Problem-focused coping	*0.80*	0.41 **	0.02	0.38 **
2. Socially supported emotion-focused coping		*0.73*	0.09	0.30 **
3. Avoidant coping			*0.62*	0.46 **
4. Passive coping				*0.58*

*Note.* ** *p* < 0.01. Cronbach alphas are presented on the main diagonal and printed in italic.

## Data Availability

The data are available upon request to: office@psychosocialinnovation.net.
